# *Lactobacillus paragasseri* as a novel causative pathogen of cavernosal abscess

**DOI:** 10.1016/j.idcr.2021.e01320

**Published:** 2021-10-27

**Authors:** Hirokazu Toyoshima, Takuji Shibahara, Motoaki Tanigawa, Naoto Masuda, Chiaki Ishiguro, Hiroyuki Tanaka, Yuki Nakanishi, Shigetoshi Sakabe

**Affiliations:** a Department of Infectious Diseases, Japanese Red Cross Ise Hospital, 1-471-2, Funae, Ise, Mie 516-8512, Japan; b Department of Urology, Japanese Red Cross Ise Hospital, 1-471-2, Funae, Ise, Mie 516-8512, Japan; c Department of Respiratory Medicine, Japanese Red Cross Ise Hospital, 1-471-2, Funae, Ise, Mie 516-8512, Japan; d Department of Medical Technology, Japanese Red Cross Ise Hospital, 1-471-2, Funae, Ise, Mie 516-8512, Japan

**Keywords:** *Lactobacillus paragasseri*, Cavernosal abscess, Foreign body, Microbial biochemical examination, Matrix-assisted laser desorption ionization, 16S rRNA sequencing

## Abstract

Most cases of cavernosal abscesses result from the progression of sexually transmitted diseases (STDs) commonly caused by *Neisseria gonorrhoeae*; however, cavernosal abscesses without STDs are rare events. Herein, we describe the first case report of a *Lactobacillus paragasseri* cavernosal abscess. A 63-year-old man with diabetes and a history of foreign object insertion into the urethra one year prior presented with high-grade fever and slight local pain. The patient was diagnosed with *L*. *paragasseri* cavernosal abscess based on computed tomography and microbial biochemical examinations in addition to matrix-assisted laser desorption ionization (MALDI) and 16S rRNA sequencing from blood and pus cultures. The patient was successfully treated with a surgical procedure and appropriate antimicrobials. Hence, *L*. *paragasseri*, a commensal bacterium of the gastrointestinal or genitourinary tract, can cause cavernosal abscesses. Notably, *L*. *paragasseri* is difficult to distinguish from *L*. *gasseri* using MALDI or 16S rRNA sequencing without microbial biochemical examinations owing to morphological similarities. Therefore, special attention should be paid, clinically and microbiologically, to the potential of *L*. *paragasseri* cavernosal abscess in clinical settings.

## Highlights

• *L*. *paragasseri* causes oligosymptomatic febrile spontaneous cavernosal abscesses.
• Molecular identification methods alone can misidentify *L*. *paragasseri* as *L*. *gasseri.*
• Molecular and microbial biochemistry methods can identify *L*. *paragasseri.*
• Penicillins are optimal antimicrobials for treatment of *L*. *paragasseri* infections.
• *L*. *paragasseri* cavernosal abscesses need drainage with appropriate antimicrobials.

## Introduction

*Lactobacillus* species are gram-positive, non-sporing, and non-motile rods, which are commensals of the gastrointestinal and genitourinary tract. *Lactobacillus paragasseri* is often confused with *L*. *gasseri* because of their molecular similarity. *L*. *paragasseri* was first described in 2018 as a sister taxon of *L*. *gasseri* based on whole-genome sequence analyses [1]. *L*. *gasseri* causes several infections, including bacteremia, dental caries, and empyema; however, the role of *L*. *paragasseri* remains unclear. *L*. *paragasseri* can often be misidentified as *L*. *gasseri* by matrix-assisted laser desorption ionization (MALDI) or 16S rRNA sequencing that shows 99.0% similarity between *L*. *paragasseri* and *L*. *gasseri* [1]. Hence, *L*. *paragasseri* infections reported in previously published case reports could have been misidentified as *L*. *gasseri* infections in the absence of appropriate microbial biochemical examinations.

### Case report

A 63-year-old Japanese man with a history of foreign object insertion into the urethra one year prior presented with high-grade fever, general fatigue, loss of appetite, and spontaneous slight genital pain. The patient also presented with poorly managed diabetes and a left inguinal hernia.

The patient was alert, with body temperature of 40.2 °C, blood pressure 181/102 mmHg, heart rate 122 beats/min, respiratory rate 24 breaths/min, and oxygen saturation 95% in ambient air. Physical examination revealed general gingivitis, chronic periodontitis, and swelling of the area from the penile base to the scrotum with redness, warmth, and mild tenderness. Laboratory findings were as follows: white blood cell count 17,100/µL with 88.1% neutrophils, hemoglobin 14.0 g/dL, platelet count 28.0 × 10^4^/µL, albumin 3.2 g/dL, alanine transferase 26 U/L, lactate dehydrogenase 240 U/L, creatine kinase 120 U/L, blood urea nitrogen 7 mg/dL, creatinine 0.67 mg/dL, C-reactive protein 14.49 mg/dL, blood glucose 372 mg/dL, and hemoglobin A1c 11.5%.

**Fig. 1:**
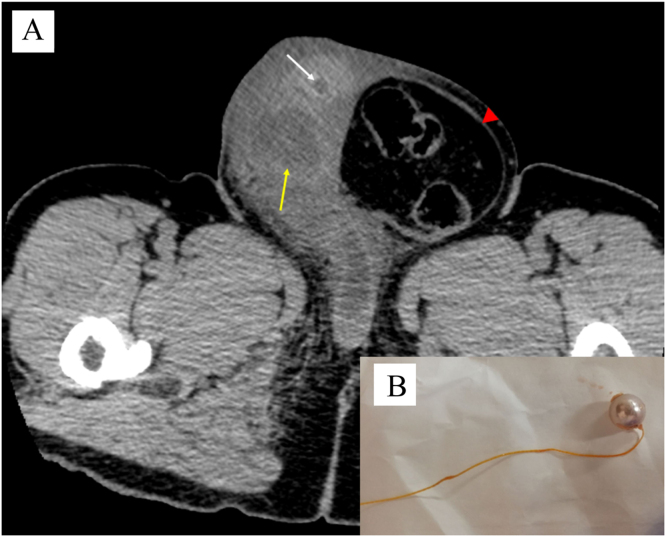
(A) Computed tomography showing a left inguinal hernia (red arrowhead), foreign body (white arrow) in the urethra, and irregular low-density area (yellow arrow), indicating an abscess in the penile corpus cavernosum. (B) The foreign body was removed and identified as a pearl with a thread.

**Fig. 2:**
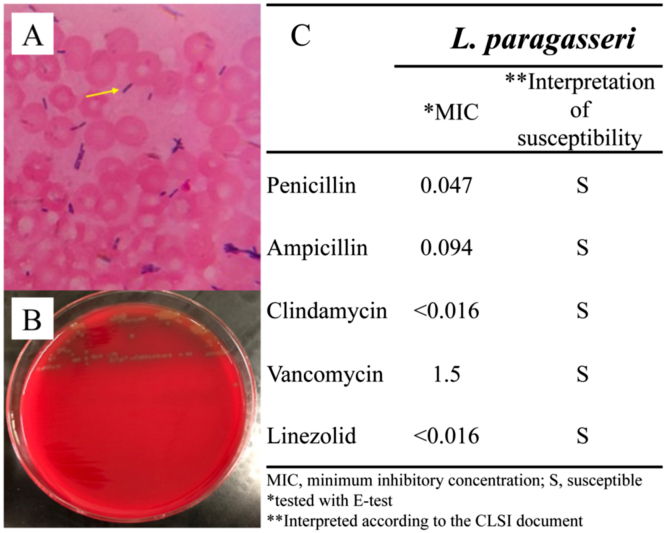
(A) Gram staining (magnification: ×1000) revealed gram-positive rods (yellow arrow). (B) The colonies were circular, flat, and pinhead, with a zone of alpha-hemolysis. (C) Susceptibility testing of the isolates from blood culture with the E-test revealed minimum inhibitory concentrations for penicillin and ampicillin of 0.047 and 0.094 µg/mL, respectively.

Computed tomography (CT) revealed a foreign body in the urethra, presenting bilateral hydronephrosis and an irregular low-density area with inflammation, which was consistent with an abscess in the corpus cavernosum (Fig. 1A). The foreign body, identified as a pearl with thread (Fig. 1B), was manually removed and a catheter was placed in the bladder to relieve urinary retention. Two sets of blood cultures were performed, and intravenous meropenem (0.5 g) and clindamycin (600 mg) were administered every 12 h. The aerobic and anaerobic blood cultures showed presence of gram-positive rods after 1-day incubation (Fig. 2A). The isolates grew as circular, flat, and pinhead colonies, with an alpha-hemolytic zone on 5% sheep blood agar after additional 2 days of incubation under 5% CO_2_ at 37 °C (Fig. 2B). The bacteria were identified as *L*. *gasseri* via MALDI with a high score of 2.32, whereas 16 S rRNA sequencing revealed the causative bacteria to be *L*. *paragasseri*, with identities of 100% (1487/1487) [2]. After additional microbial biochemical examinations, the bacteria were conclusively identified as *L*. *paragasseri*. The E-test showed penicillin and ampicillin to have minimum inhibitory concentrations of 0.047 and 0.094 µg/mL, respectively, indicative of susceptibility according to the Clinical and Laboratory Standards Institute criteria (Fig. 2C) [3].

**Fig. 3:**
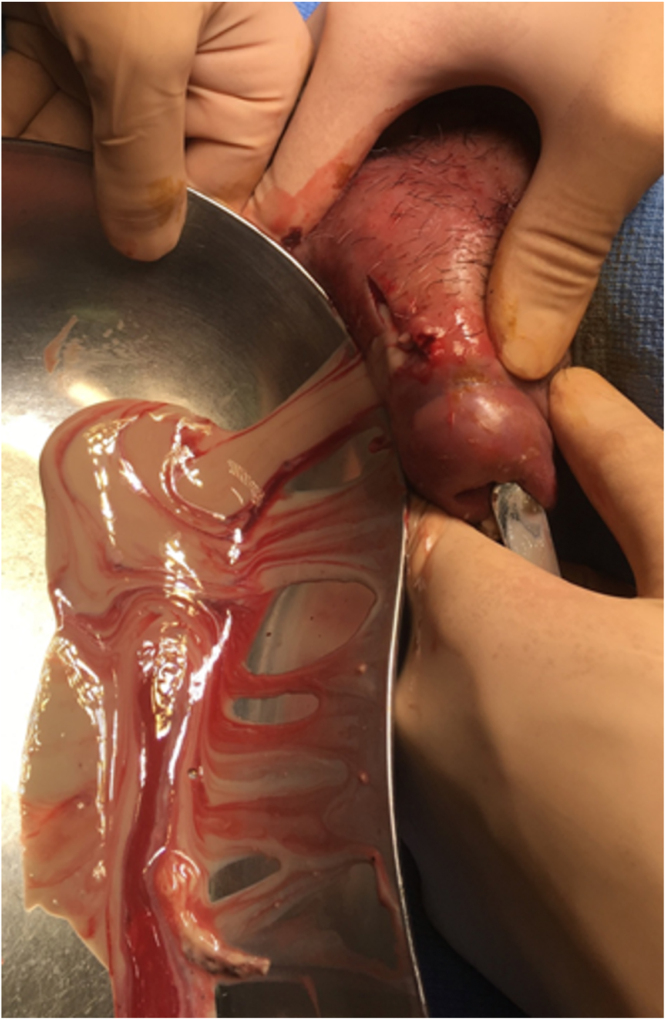
Incision of the right corpus cavernosum contributed to drainage of a large amount of pus.

**Fig. 4:**
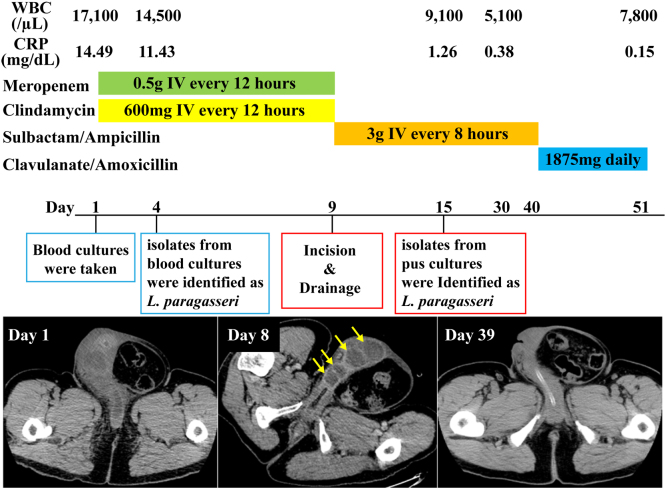
Clinical course of the patient. The patient did not improve despite the removal of the foreign body and the indwelling of the catheter. Appropriate microbiological examinations of blood cultures revealed the causative agent to be *L*. *paragasseri*. Enhanced CT imaging on day 8 revealed progression toward multilocular abscesses in the corpus cavernosum (yellow arrows). Incisional drainage of the right corpus cavernosum was performed on day 9. Pus culture also revealed *L*. *paragasseri* in the same way. Intravenous ampicillin/sulbactam was administered according to the susceptibility testing. Thereafter, ampicillin/sulbactam was changed to oral amoxicillin/clavulanate on day 40 confirming reduced abscess size by CT imaging on day 39. Consequently, oral amoxicillin/clavulanate was discontinued, confirming the disappearance of the abscess without recurrence and sequelae.

Despite the antimicrobial therapy, the high-grade fever persisted, and the local pain was exacerbated. Enhanced CT on day 8 revealed progression toward multilocular abscesses in the corpus cavernosum. Therefore, the antimicrobials were switched to ampicillin/sulbactam (3 g every 8 h) based on the susceptibility testing. Furthermore, incisional drainage of the right corpus cavernosum was performed on day 9 (Fig. 3). The patient became apyrexial after the invasive procedure and antimicrobial change. Pus culture revealed the presence of *L*. *paragasseri* with consistent susceptibilities. Thereafter, ampicillin/sulbactam was changed to oral amoxicillin/clavulanate (1875 mg daily). On day 40, the reduced abscess size was confirmed by CT findings, and amoxicillin/clavulanate was discontinued. Consequently, the patient remained disease-free without recurrence or sequelae during a 2-year follow-up period (Fig. 4).

## Discussion

This report illustrates three main clinical issues: the potential of *L*. *paragasseri* to cause cavernosal abscess in a clinical setting, impaired capacity to distinguish *L*. *paragasseri* from the similar species *L*. *gasseri*, and a potential treatment strategy for *L*. *paragasseri*-induced cavernosal abscess.

*L*. *gasseri* is widely recognized as a commensal bacterium inhabiting the gastrointestinal and genitourinary tracts and can cause several infections, such as bacteremia, dental caries, empyema, and peritonitis [4], [5], [6], [7]. However, to date, no reports have described cavernosal abscesses caused by *L*. *paragasseri*. Cavernosal abscesses can occur either spontaneously, or following intracavernosal injection, trauma, or foreign body insertion [8]. In the present case, odontogenic infections and the urethral foreign body may have led to the onset and exacerbation of the *L*. *paragasseri*-triggered cavernosal abscess. Cavernosal abscesses are commonly caused by *Staphylococcus aureus*, streptococci, *Bacteroides* and *Fusobacterium* species [9]; however, the most common pathogen is *Neisseria gonorrhoeae,* with infections generally occurring secondary to urethritis caused by sexually transmitted diseases (STDs) [8]. These microorganisms are easily identified using gram staining, leading to appropriate antimicrobial therapy based on local antibiograms. However, this report highlights that clinicians should pay attention to the potential of *L*. *paragasseri* involvement in cavernosal abscess cases with gram-positive rods identified by gram staining of pus or blood cultures.

Several strains, including the Japanese Collection of Microorganisms (JCM) 5343, JCM 5344, and JCM 1130, have been identified as *L*. *gasseri* American Type Culture Collection (ATCC) 33323. These strains show a 99.9% similarity to *L*. *gasseri* ATCC 33323 [1]. However, the average nucleotide identity and *in silico* DNA-DNA hybridization values of these three strains compared to *L*. *gasseri* ATCC 33323 were less than the widely accepted threshold to distinguish strains [1]. Therefore, JCM 5343, JCM 5344, and JCM 1130 were reclassified as *L*. *paragasseri* [1]. Moreover, these strains have different microbial biochemical characteristics compared with those of *L*. *gasseri* [1]. In this study, the isolate was identified as *L*. *paragasseri* JCM 5343 via 16S rRNA sequencing based on the GenBank Basic Local Alignment Search Tool database (www.ncbi.nlm.nih.gov/genbank/) (Identities: 1487/1487; Gaps: 0/1487; Score: 2747 bits). In contrast, 16S rRNA sequencing also indicated *L*. *gasseri* ATCC 33323 with 99.0% identity (1485/1487). We confirmed *L*. *paragasseri* by performing microbial biochemical examinations, including API 50 CH assay (bioMérieux) (Table 1) and growing the culture in the presence of NaCl. The findings were not consistent with the characteristics of *L*. *gasseri*, but instead with those of *L*. *paragasseri* JCM 5343, despite the identification of *L*. *gasseri* by MALDI.

**Table 1:** Characterization of the microbial biochemical parameters.

	The isolate in this case	*L*. *paragasseri* JCM 5343	*L*. *paragasseri* JCM 5344	*L*. *paragasseri* JCM 1130	*L*. *gasseri* JCM 1131	*L*. *gasseri* JCM 1025
D-Ribose	˗˗	˗˗	˗˗	˗˗	˗˗	˗˗
D-Galactose	+	+	+	+	+	+
L-Sorbose	˗˗	˗˗	˗˗	˗˗	˗˗	˗˗
L-Rhamnose	˗˗	˗˗	˗˗	˗˗	˗˗	˗˗
D-Mannitol	˗˗	˗˗	˗˗	˗˗	˗˗	˗˗
Methyl ɑ-D-glucopyranoside	˗˗	˗˗	+	˗˗	˗˗	˗˗
N-Acetyl-glucosamine	+	+	+	+	+	+
Amygdalin	+	+	+	+	+	+
Arbutin	+	+	+	+	+	+
Salicin	+	+	+	+	+	+
Cellobiose	+	+	+	+	+	+
Lactose	+	+	+	+	+	+
Melibiose	˗˗	˗˗	˗˗	˗˗	+	+
Trehalose	+	+	+	+	+	+
Melezitose	˗˗	˗˗	˗˗	˗˗	˗˗	˗˗
Raffinose	˗˗	˗˗	˗˗	˗˗	+	+
Starch	+	+	+	+	+	˗˗
Gentiobiose	+	+	+	+	+	+
Turanose	˗˗	˗˗	+	˗˗	+	+
D-Tagatose	+	+	+	+	+	+
Growth with 2% NaCl (w/v)	+	+	+	+	+	+
Growth with 4% NaCl (w/v)	˗˗	˗˗	˗˗	+	+	+
Growth with 5% NaCl (w/v)	˗˗	˗˗	˗˗	˗˗	+	+

Abbreviation: JCM, Japanese Collection of Microorganisms.

The optimal antimicrobial therapy for *L*. *paragasseri* cavernosal abscesses has not been standardized owing to a lack of relevant of studies. However, most strains of *L*. *gasseri* are susceptible to penicillin and ampicillin [4]. Additionally, it is likely that previous cases of *L*. *paragasseri* infection were erroneously identified as *L*. *gasseri*, as *L*. *paragasseri* was first described in 2018 [1]. Our patient was successfully treated with ampicillin/sulbactam, which suggests that penicillin in an optimal antimicrobial for *L*. *paragasseri* infections. In general, anaerobes, including *Bacteroides* and *Fusobacterium* species, can cause cavernosal abscesses. Given the potential of anaerobic coinfection, ampicillin/sulbactam was administered as a definitive therapy because anaerobic transport devices and chambers were not available in our hospital. The optimal duration of antimicrobial therapy still needs to be optimized. Cavernosal abscesses may recur, and a case of total penectomy necessitated due to abscess recurrence has been reported [10], [11]. In this case, the patient, who had poorly managed diabetes, progressed to multilocular abscesses despite removal of the foreign body and intravenous antimicrobial therapy. Hence, penicillins, to which *L*. *paragasseri* was susceptible, were continued for 6 weeks after the switch was made from the previous antimicrobials along with surgical incision and drainage. This treatment contributed to the disappearance of the abscess as confirmed by CT imaging, and no recurrence or sequelae were observed.

Surgical incision and drainage combined with appropriate antimicrobial administration should be considered as first-line treatment in such cases [9]; however, this approach can result in postoperative complications, such as penile deviation or erectile dysfunction [9]. Therefore, less invasive interventional techniques (e.g., image-guided aspiration) are performed in some cases [10]. In our case, surgical incision and drainage were performed, considering the difficulty of appropriate drainage because of multilocular abscesses.

In conclusion, oligosymptomatic cavernosal abscesses caused by *L*. *paragasseri* may occur without STDs. Additionally, *Lactobacillus* species present as gram-positive rods, which are distinct from microorganisms that commonly cause cavernosal abscesses, such as *N. gonorrhoeae*, *S. aureus*, streptococci, *Bacteroides* and *Fusobacterium* species. *L*. *paragasseri* can be identified using a combination of molecular and microbial biochemical examinations, despite its similarity to *L*. *gasseri*. Moreover, penicillins are optimal antimicrobials for *L*. *paragasseri* infections. Clinicians should pay special attention to the accurate identification and appropriate antimicrobial therapy for *L*. *paragasseri* infections.

## Authors’ contributions

H Toyoshima contributed to the clinical management of the patient and was involved in study conception, acquisition, and analysis of the data, and drafting of the manuscript. TS contributed to the clinical management of the patient and was involved in the acquisition and analysis of the data. NM and CI acquired and analyzed the data. H Tanaka and YN conceived the study. MT and SS supervised manuscript preparation and revisions. All authors reviewed the final draft of the manuscript and have approved its submission.

## Consent

Written informed consent was obtained from the patient for the publication of this case report and accompanying images. A copy of the written consent is available for review by the Editor-in-Chief of this journal on request.

## Ethical approval

This study was approved by the institutional review board and ethics committee of the Japanese Red Cross Ise Hospital (approval number: ER2021-11).

## CRediT authorship contribution statement

**Hirokazu Toyoshima**: Conceptualization, Methodology, Data curation, Writing - Original draft, Writing - Review and Editing, Visualization. **Takuji Shibahara**: Methodology, Data curation. **Motoaki Tanigawa**: Supervision. **Naoto Masuda**: Conceptualization, Methodology. **Chiaki Ishiguro**: Conceptualization, Methodology. **Hiroyuki Tanaka**: Methodology. **Yuki Nakanishi**: Methodology. **Shigetoshi Sakabe**: Supervision.

## Disclosure statement

The authors state that they have no conflicts of interest. This research did not receive any specific grant from funding agencies in the public, commercial, or not-for-profit sectors.
